# AcidoCEST-UTE MRI Reveals an Acidic Microenvironment in Knee Osteoarthritis

**DOI:** 10.3390/ijms23084466

**Published:** 2022-04-18

**Authors:** Alecio F. Lombardi, Yajun Ma, Hyungseok Jang, Saeed Jerban, Qingbo Tang, Adam C. Searleman, Robert Scott Meyer, Jiang Du, Eric Y. Chang

**Affiliations:** 1 Research Service, Veterans Affairs San Diego Healthcare System, San Diego, CA 92161, USA; q1tang@health.ucsd.edu (Q.T.); ericchangmd@gmail.com (E.Y.C.); 2 Department of Radiology, University of California San Diego, San Diego, CA 92161, USA; yam013@health.ucsd.edu (Y.M.); h4jang@health.ucsd.edu (H.J.); sjerban@health.ucsd.edu (S.J.); asearleman@health.ucsd.edu (A.C.S.); jiangdu@health.ucsd.edu (J.D.); 3 Orthopaedic Surgery Service, Veterans Affairs San Diego Healthcare System, San Diego, CA 92161, USA; robert.meyer3@va.gov

**Keywords:** CEST, MRI, UTE, chemical exchange saturation transfer, ultrashort echo time, cartilage, meniscus, pH, osteoarthritis, OA

## Abstract

A relationship between an acidic pH in the joints, osteoarthritis (OA), and pain has been previously demonstrated. Acidosis Chemical Exchange Saturation Transfer (acidoCEST) indirectly measures the extracellular pH through the assessment of the exchange of protons between amide groups on iodinated contrast agents and bulk water. It is possible to estimate the extracellular pH in the osteoarthritic joint using acidoCEST MRI. However, conventional MR sequences cannot image deep layers of cartilage, meniscus, ligaments, and other musculoskeletal tissues that present with short echo time and fast signal decay. Ultrashort echo time (UTE) MRI, on the other hand, has been used successfully to image those joint tissues. Here, our goal is to compare the pH measured in the knee joints of volunteers without OA and patients with severe OA using acidoCEST-UTE MRI. Patients without knee OA and patients with severe OA were examined using acidoCEST-UTE MRI and the mean pH of cartilage, meniscus, and fluid was calculated. Additionally, the relationship between the pH measurements and the Knee Injury and Osteoarthritis Outcome Score (KOOS) was investigated. AcidoCEST-UTE MRI can detect significant differences in the pH of knee cartilage, meniscus, and fluid between joints without and with OA, with OA showing lower pH values. In addition, symptoms and knee-joint function become worse at lower pH measurements.

## 1. Introduction

Osteoarthritis (OA) is a major cause of years lived with a disability [1] and increased health costs worldwide [2], which has shown a steady increase in incidence rate in the past three decades [3,4]. Despite many research efforts focusing on new treatments, the results have been limited and patients rely largely on symptomatic or rehabilitation therapies [4].

Imaging of OA has seen considerable developments in the last two decades, including the validation of high-resolution and three-dimensional MR sequences for the evaluation of cartilage thickness and volume [5,6], the use of nuclear medicine as a potential biomarker of early and progressive OA [7,8], the development of compositional MRI analysis for the characterization of cartilage matrix such as T2 mapping, T1ρ mapping [6,7,8,9,10], and glycosaminoglycan Chemical Exchange Saturation Transfer (gagCEST) [11,12], among others.

One interesting method of cartilage analysis is the acidosis Chemical Exchange Saturation Transfer (acidoCEST) technique, which measures the exchange of protons between amide groups on iodinated contrast agents and bulk water [13]. The rate of this chemical exchange is proportional to the extracellular pH of the tissue being studied [13]. A relationship between an acidic pH in the joints, OA, and pain has been demonstrated in some studies. Konttinen et al., for instance, found that the pH of normal cartilage was higher than the pH of damaged cartilage using an intraoperative sting electrode [14]. Deval et al. showed that acid-sensing ion channels (ASICs) expressed in central and peripheral nervous systems and activated by extracellular acidosis are strongly correlated with nociceptor excitability and pain [15], whereas Izumi et al. reported that acidic-sensing ion channels type 3 (ASIC3) expressed on primary afferent fibers innervating joints are strongly associated with weight-bearing pain and secondary hyperalgesia in animal osteoarthritic models [16].

One potential issue, however, is that the deep and calcified layers of cartilage are devoid of signals on conventional MR sequences. This cartilage region may be important, though, especially in the early development of OA [17]. Ultrashort echo time (UTE) sequences, on the other hand, are of great interest as they can acquire signals from deep layers of cartilage and other musculoskeletal tissues with low T2 relaxation times [18]. Mahar et al., for example, showed that fast-spin echo (FSE) MR sequences can measure cartilage relaxation times to a depth of approximately 460 µm, whereas UTE MR sequences can reliably measure relaxation times to a depth of approximately 700 µm. Furthermore, recent developments of UTE sequences have enabled image acquisition of the deep layers of cartilage with high contrast and resolution in an acceptable scan time [19,20], which will be further shortened as advanced acceleration techniques become integrated [21].

Previously, Ma et al. used acidoCEST-UTE MRI to measure pH in liquid and tissue phantoms and showed a strong correlation with the pH measured using electrodes [22]. Both iopamidol and iohexol were promising agents over a pH range of 6.2 to 7.8 [22]. High et al. later showed that acidoCEST-UTE MRI using either iopamidol or iohexol could be used for in vivo pH measurements with comparable values, again using electrodes as a reference [23]. The main goal of this current study is to compare the pH measured in the knee joints of volunteers without OA (as defined by the American College of Rheumatology) [24] and patients with severe OA using acidoCEST-UTE MRI. Our secondary goal is to evaluate the correlations between the pH measurements and the Knee Injury and Osteoarthritis Outcome Score (KOOS). We hypothesize that the pH will be lower/acidic in patients with knee OA and that the pH measurements will be strongly correlated with the KOOS.

## 2. Results

### 2.1. Participants, Demographics, and KOOS Score Comparisons among Groups

Sixteen patients were enrolled in the study, including nine without OA (all males, mean age 48 ± 16 years) and seven patients with severe OA (six males and one female, mean age 65 ± 8 years). Table 1 shows the demographics of all participants as well as the mean KOOS score in each subscale and the mean visual analog pain score for each group of participants.

All the KOOS subscale scores showed significantly lower values in patients with OA compared with patients without OA (*p* < 0.001) (Table 1). The visual analog pain scale scores were significantly higher in patients with OA (*p* < 0.001) (Table 1).

### 2.2. pH Measurements

In total, 448 image slices (16 knee scans × 28 sagittal slices) were processed using the MATLAB code framework, yielding one final mean pH value for cartilage, meniscus, and fluid for each patient.

Considering all ROIs together, the mean pH in patients with OA (6.40 ± 0.08) was significantly lower than the mean pH in patients without OA (7.01 ± 0.26, *p* < 0.001).

**Figure 1:**
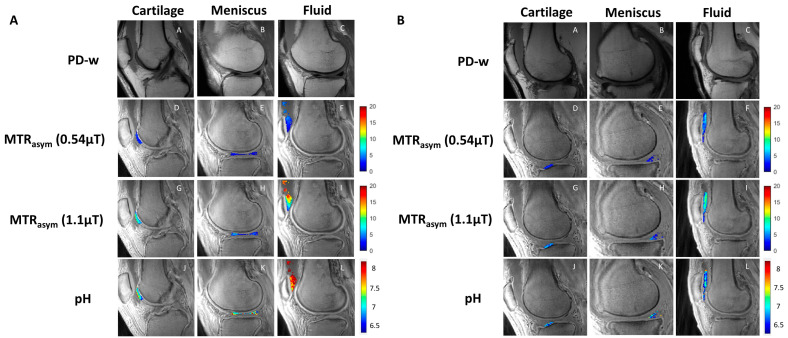
Representative image examples from patients without OA (**A**) and with OA (**B**). Sagittal PD-weighted (first row), low-power acido-CEST UTE (second row), high-power acido-CEST UTE (third row), and pH pixel maps (fourth row) of cartilage, meniscus, and fluid. The pH is directly correlated with the radiofrequency power mismatch (RPM) measurements, as described in Equations (3) and (4). Note the higher pH values (yellow and red colors) in patients without OA compared with patients with OA (blue colors).

Figure 1 shows examples of the resultant magnetization transfer ratio asymmetry (MTRasym) and pH pixel maps from patients without OA (Figure 1A) and patients with OA (Figure 1B), with ROIs drawn in the cartilage, meniscus, and fluid and the respective comparisons with PD-weighted MR images.

**Figure 2:**
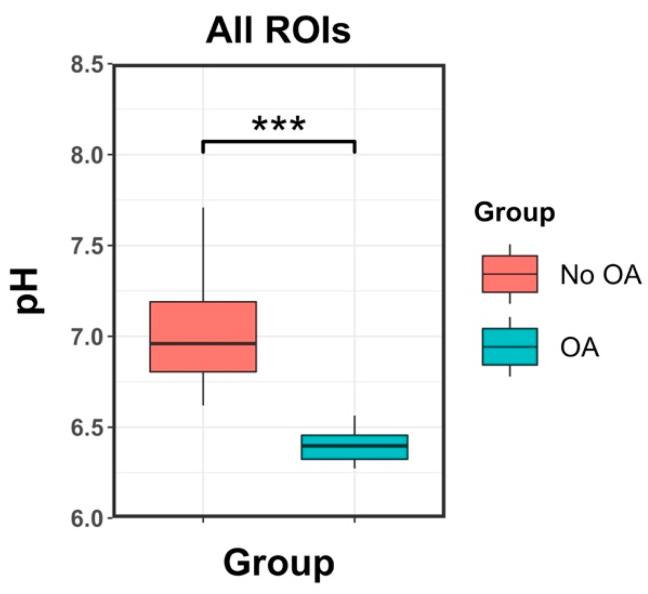
Boxplots of mean pH measurements versus groups for all ROIs. Significant differences are observed between the two groups. ***: *p* values lower than 0.001.

Figure 2 shows a boxplot of pH versus the group of patients when considering all ROIs together.

When taking into consideration each ROI type separately, the mean pH of cartilage, meniscus, and fluid were all significantly lower in patients with OA compared with patients without OA (*p* < 0.01 for cartilage, *p* = 0.015 for meniscus, and *p* < 0.001 for fluid).

**Figure 3:**
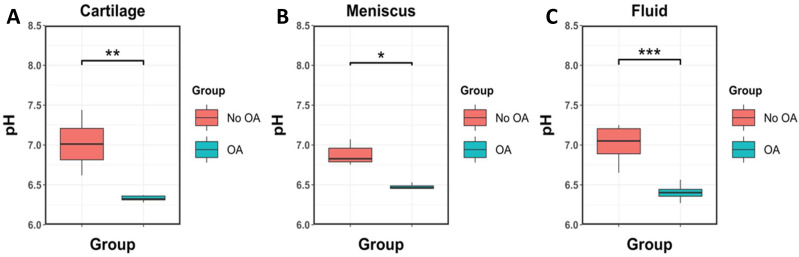
Boxplots of mean pH measurements versus each group of participants (No OA and OA) for ROIs drew in cartilage (**A**), meniscus (**B**), and fluid (**C**). Significant differences in pH measurements are observed between groups with lower pH in patients with OA compared with patients without OA. “***”: *p* values lower than 0.001; “**”: *p* values lower than 0.01; “*”: *p* values lower than 0.05.

Figure 3 shows boxplots of pH versus groups considering each ROI type separately (i.e., cartilage, meniscus, and fluid).

When considering different ROI types within the same group (i.e., no OA patients and OA patients), no significant differences were observed in the mean pH between cartilage, meniscus, and fluid, except meniscus versus cartilage in the OA group, where the meniscus showed a higher pH (*p* = 0.024).

**Figure 4:**
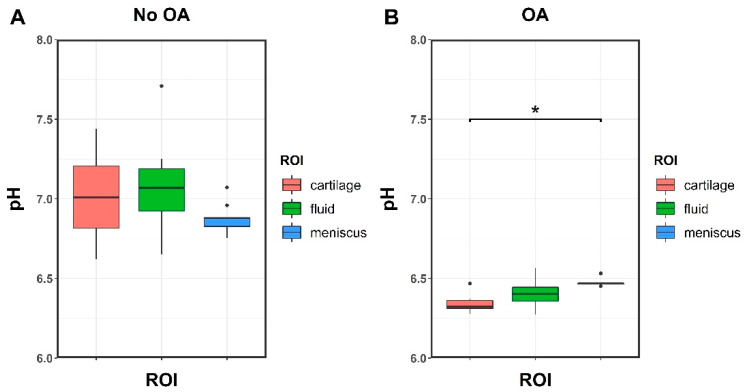
Boxplots of mean pH measurements versus ROIs in patients without OA (**A**) and with advanced OA (**B**). No significant differences were found, except for the pH of cartilage and meniscus in the OA group (*p* = 0.024). “*”: *p* values lower than 0.05; “•”: outliers.

Box plots of pH measurements versus ROIs for each group can be seen in Figure 4.

### 2.3. Correlations between the pH Measurements and the KOOS Score

All the subscales of the KOOS score showed strong direct correlation coefficients with mean pH measurements in both study groups, varying from 0.61 in the patellofemoral subscale to 0.72 in the pain subscale (*p* < 0.001). The visual analog pain scale showed a strong inverse correlation coefficient with mean pH measurements (R = – 0.83, *p* < 0.001).

**Figure 5:**
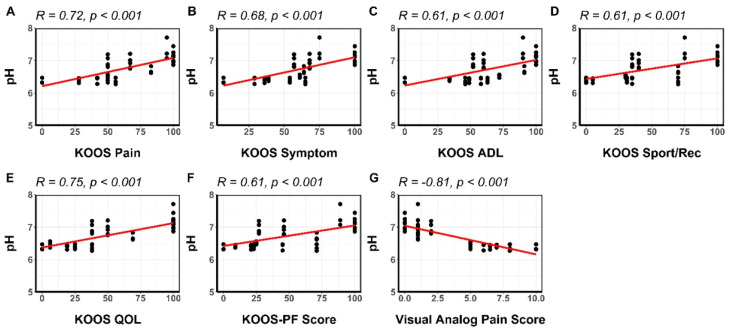
Scatterplots of pH versus KOOS scores and the visual analog pain score. Strong direct correlations were observed for all KOOS subscale scores (**A**–**F**). There was a strong inverse correlation between pH measurements and the visual analog pain score (**G**). KOOS: Knee Injury and Osteoarthritis Outcome Score; ADL: activities of daily living; Sports/Rec: sports and recreation activities; QOL: quality of life; PF: patellofemoral.

Figure 5 shows the scatterplots and the respective correlation coefficients for each subscale of the KOOS score and the visual analog pain scale plotted against the mean pH throughout all ROIs.

## 3. Discussion

In this study, we investigated pH measurements of cartilage, meniscus, and fluid using acidoCEST-UTE MRI in the knee joints of patients without OA and with advanced OA. We also correlated the pH measurements with the KOOS and visual analog pain scores from all participants. The pH was significantly lower in patients with advanced OA compared with patients without OA. We also found a strong direct correlation between pH measurements and all the KOOS subscales, as well as a strong inverse correlation between pH and the visual analog pain scale.

While T2 mapping offers an estimate of cartilage’s water content [25] and T1ρ mapping offers an estimate of cartilage’s proteoglycan content [26], other biomarkers may be useful to predict early OA degeneration and progression. For example, glycosaminoglycan (GAG) CEST may estimate the GAG content in cartilage in patients with OA [12,27,28,29]. Additionally, delayed gadolinium-enhanced MRI of cartilage (dGEMRIC) measures are strongly correlated with cartilage’s proteoglycan content [30], and with the development of joint-space narrowing [31].

We now demonstrate that pH can be measured in OA, confirming findings from prior studies that have investigated the role of pH in OA development. Our study is following Konttinen et al.’s results in which the investigators assessed intraoperative measurements of pH of clinically normal, fibrillated, superficially fissured, and deeply fissured cartilage in OA patients undergoing hip replacement using sting electrodes and compared it with the expression of cathepsin K, an acid-activated collagenase, in the same harvested tissue [14]. Cathepsin K is an acidic cysteine endoproteinase that is activated and degrades collagen at pH 4.5 and 6.0 [32], by cleaving the collagen I/II triple helix in its N-terminal [33]. This is distinct from collagenase-1 (MMP-1), collagenase-2 (MMP-8), collagenase-3 (MMP-13), and stromelysin-1, which can also cleave and solubilize the collagen superhelix, but work preferentially in neutral pH [34]. Konttinen et al. hypothesized that cathepsin K is produced by phenotypically altered chondrocytes, which may initiate or accelerate cartilage degeneration. Indeed, in their study, the pH of normal cartilage was 7.1 ± 0.4, compared with 6.2 ± 0.9, 5.7 ± 1.0, and 5.5 ± 1.0 for grades 1–3 cartilage degeneration, respectively, possibly indicating a progressive presence or effect from normal through more degenerated cartilage. They also found that cathepsin K was overexpressed in OA joints using acridine orange staining, quantitative reverse-transcriptase-polymerase chain reaction (RT-PCR), immunohistochemistry, antigen preabsorption, and gel electrophoresis with immunoblotting analysis. They concluded that acid-activated cathepsin K was induced in OA, stressing the intimate relationship between an acidic microenvironment and cartilage degeneration. Our results show similar values for cartilage pH in joints without and with OA measured using acidoCEST-UTE MRI. Our lowest cartilage pH values were higher than more acidic and degenerated cartilage pH values, probably because our workflow is only calibrated to perform well within the pH range of 6.2 to 7.8, but it does not exclude the possibility of lower pH values in our patients. Future improvements in the sequence parameters, post-processing, and use of greater magnetic field strengths may result in an extension of this range.

Moreover, there is mounting evidence that acidification plays an important role in pain. Acid-sensing ion-channel receptors (ASICs) present in the dorsal root ganglion are responsible for hyperalgesia in joint inflammation [16,35]. Izumi et al. found that ASIC type 3 in afferent neural fibers originating from joints is strongly associated with weight-bearing pain in an OA animal model [16]. They found that ASIC3 was significantly upregulated after the induction of OA and that animals presented not only primary but also secondary hyperalgesia. Initial joint inflammation may reduce local pH, resulting in the upregulation of ASIC3. Some studies show that extracellular acidification can induce chondrocyte apoptosis [36] and that the inhibition of ASICs may protect against chondrocyte injury [37].

AcidoCEST MRI has been studied mostly for the evaluation of tumors [13,38,39]. Few studies have tried to use acidoCEST to assess the pH of cartilage or other musculoskeletal tissues [22,23]. One technical challenge is to acquire signals from very short T2 species such as the meniscus or deep layers of cartilage, which can be overcome by UTE MRI sequences. Previous studies validated the use of acidoCEST-UTE MRI for the pH measurements of cartilage, meniscus, and fluid in phantoms using either iopamidol or iohexol, and showed initial feasibility for in vivo translation [22,23]. Our current results show that acidoCEST-UTE MRI can also show significant differences in the pH of the knee joints between patients without and with osteoarthritis, with strong correlations between the pH measurements, symptoms, and joint function. Future potential applications of acidoCEST-UTE MRI may be in the research of response to treatment using new techniques that rely on inflammatory, enzymatic, and metabolic changes in cartilage, such as new anti-inflammatory drugs [40], the intra-articular injection of stem-cells [41], or the intra-articular injection of platelet-rich plasma [42].

Our study has limitations. First, we had a relatively small sample size, though the differences in pH measurements between groups and correlations were substantial enough to reach statistical significance. Second, the majority of our patients were male, but this reflects the cohort of patients at our institution (90% are males). Third, the fluid pH measurements in our study should be interpreted with caution. We did not test the pH values of the injected contrast agents, which according to the manufacturer, can range between 6.5 and 7.7. This contrast mixes with the existing fluid in the joint environment, and several prior studies have confirmed that synovial fluid pH is lower in osteoarthritic [43] and inflammatory arthritis [44,45]. Fourth, further studies are needed to estimate cutoff points of pH measurements between normal and abnormal patients and to elucidate the biological mechanisms. Fifth, the success of acidoCEST MRI depends on the contrast agent uptake into the tissues being studied, and the spectra may be contaminated by chemical exchanges not related to the contrast agent, such as from other surrounding protons or the nuclear Overhauser effect. Sixth, we did not include in vivo measurements of joint tissue pH due to a lack of suitable FDA-approved pH meters. Finally, another limitation is that acidoCEST-UTE MRI requires multiple frequency offset acquisitions that increase the total scan time. However, this could be shortened considerably as acceleration techniques become integrated, such as deep learning reconstruction.

## 4. Materials and Methods

### 4.1. Volunteers and Patients

This prospective study was approved by our institutional review board (IRB approval number H170124) and all participants signed informed consent. Patients presenting to the department of radiology were consecutively recruited by a musculoskeletal radiologist with the following inclusion criteria: no knee symptoms or having a Kellgren and Lawrence grade of 0–1 on knee radiographs obtained the same day, willingness to undergo the entire acidoCEST-UTE MRI examination, and no contraindications to iodinated contrast or MRI. For the group with severe OA, patients scheduled for total knee arthroplasty were consecutively recruited by an orthopedic surgeon with the following inclusion criteria: knee radiographs with Kellgren and Lawrence grade 4 OA, willingness to undergo the entire acidoCEST-UTE MRI examination, and no contraindications to iodinated contrast or MRI. For each patient, a 20-gauge needle was inserted into the knee joint through an anterior approach under fluoroscopic imaging guidance. A total of 20–25 cc of contrast was injected into the joint (*n* = 3, iopamidol 370 mgI/mL [Bracco Imaging S.p.A., Milan, Italy]; *n* = 13, iohexol 350 mgI/mL [GE Healthcare, Chicago, IL, USA]) and imaging was performed approximately 1 h after injection. All participants were asked to respond to the Knee Injury and Osteoarthritis Outcome Score (KOOS) as well as to grade their knee pain using a visual analog scale.

### 4.2. Imaging with Acidocest-UTE MRI

MRI scanning was performed on a 3T clinical MRI scanner (MR750, GE Healthcare, Milwaukee, WI, USA) using an 8-channel transmit/receive knee coil. The CEST pulse sequence consists of a frequency-selective Fermi saturation pulse (duration = 32 ms, bandwidth = 40 Hz) followed by the acquisition of a 3D ultrashort echo time (UTE) cones sequence. The imaging parameters were: TR = 62 ms, TE = 0.032 ms, number of spokes (Nsp) = 5, spoke interval (τ) = 5 ms, flip angle (FA) = 5°, bandwidth (BW) = 125 kHz, field of view (FOV) = 13 × 13 × 8.4 cm, and matrix size = 236 × 236 × 28. Z-spectra were acquired at varying saturation offset frequencies from ± 430 Hz to ± 670 Hz with a step size of ± 40 Hz using two different saturation pulses with B1s of 0.54 and 1.10μT as calculated by power average [46]. The scanning time of each frequency was 2 min. Acquisitions were interleaved (e.g., +/− ppm and 0.54/1.10 μT powers for each offset were paired). A dual-echo UTE sequence with two echo times (TE1 = 0.032 ms; TE2 = 2.2 ms) was performed to generate a ΔB0 map, which was used for B0 correction for CEST data processing. Standard sagittal PD-weighted and T2-weighted FSE MR sequences were performed for the characterization of cartilage defects and meniscus tears and to assist in the image segmentation. The total scan time was approximately 45 min.

### 4.3. Data Processing and pH Calculations

Data were processed using MATLAB (Mathworks, Natick, MA, USA). Images were assessed by an experienced radiologist for motion, and if detected, the Elastix motion correction algorithm was applied [47]. CEST images with distinct frequency offsets were Gaussian-smoothed, interpolated, and B0-corrected pixel-by-pixel using ΔB0 maps. Z spectra plots were generated using the signal intensity measured in each of the frequency offsets plotted against the frequency offsets expressed in parts per million (ppm). The water resonance frequency was set to be zero, frequencies on the left side of the plot were considered negative, and frequencies on the right side of the plot were positive. The magnetization transfer ratio asymmetry (MTRasym) was calculated by the relationship between differences in signal intensities ( I ) on negative and positive frequency offsets (Δω) during the application of saturation pulses and signal intensities without saturation pulses ( Iο ):

(1) MTRasym=(I(−Δω)−I(Δω))/Iο

Radiofrequency power mismatch (RPM) was determined by the relationship between the MTRasym index in two different radiofrequency saturation pulses (RF1 and RF2):

(2) $$RPM=\frac{{\left[\left(1-MTRasym\right)/MTRasym\right]}_{RF1}}{{\left[\left(1-MTRasym\right)/MTRasym\right]}_{RF2}}$$

The CEST effect/peak is expected to occur at frequency offsets of +4.2 and +5.6 ppm for iopamidol and +4.3 ppm for iohexol. For this study, RPM at +4.2 was used for iopamidol and RPM +4.3 was used for iohexol. The pH was calculated with equations generated by best fit lines using liquid and tissue phantoms, according to results from previous studies [22,23].

For iopamidol:

(3) pH= (RPM+10.21)/(1.88)

For iohexol:

(4) pH=  (RPM+8.39)/(1.56)

Regions of interest (ROIs) were drawn over all cartilage, meniscus, and fluid regions on each PD-weighted MR image for all slices available from all participants by an experienced musculoskeletal radiologist. Pixels that had adequate CEST effects were displayed and included in the analysis. Specifically, adequate CEST effect was defined as pixels with positive MTRasym and increasing MTRasym at higher powers (e.g., pixels where greater MTRasym at the lower power compared with higher power were considered noise and excluded).

### 4.4. Statistical Analysis

Descriptive statistics were performed. The Shapiro–Wilk test was used to check for data normality distribution and the best statistical test was chosen based on the data distribution. Wilcoxon signed-rank test was used to compare the mean pH between different participants and groups. Spearman’s correlation coefficient was used to assess the relationship between the pH measurements and the KOOS score. A *p* value less than 0.05 was considered statistically significant. Analyses were performed using R v.4.0.2 (R Core Team, 2014) [48] and RStudio v.1.3.1073 (RStudio Core Team, 2020) [49].

## 5. Conclusions

In conclusion, this study showed that acidoCEST-UTE MRI can detect significant differences in the pH of knee cartilage, meniscus, and fluid between joints without and with OA, with OA showing lower pH values. In addition, we showed that symptoms and knee joint function become worse at lower pH measurements.

## Figures and Tables

**Table 1:** Participants’ demographics, KOOS score, and visual analog pain score.

Variable	All Participants	No OA	OA	*p* Value ***
Number of participants	16	9	7	
Age (years) *	57 ± 13	48 ± 16	65 ± 8	0.01
Sex **				
M	15 (93.7)	9 (100)	6 (85.7)	
F	1 (0.06)	0 (0)	1 (14.2)	
KOOS *				
KOOS pain	60 ± 19	79 ± 21	36 ± 19	<0.001
KOOS symptoms	41 ± 20	77 ± 18	41 ± 20	<0.001
KOOS ADL	46 ± 24	78 ± 19	46 ± 24	<0.001
KOOS sports/rec	24 ± 25	66 ± 29	24 ± 25	<0.001
KOOS QOL	19 ± 15	72 ± 28	19 ± 15	<0.001
KOOS PF	50 ± 34	67 ± 17	27 ± 23	<0.001
VAPS	4 ± 3	0.9 ± 0.8	6.8 ± 1.8	<0.001

OA: osteoarthritis; KOOS: Knee Injury and Osteoarthritis Outcome Score; ADL: activities of daily living; Sports/Rec: sports and recreation activities; QOL: quality of life; PF: patellofemoral; VAPS: visual analog pain score. * Data are means ± standard deviations. ** Data are the number of participants with percentages in parenthesis. *** *p* values represent the comparison between no-OA and OA groups.

## Data Availability

The data presented in this study are available on request from the corresponding author. The data are not publicly available due to the privacy of participants.
