# Correction: Lombardi et al. AcidoCEST-UTE MRI Reveals an Acidic Microenvironment in Knee Osteoarthritis. *Int. J. Mol. Sci.* 2022, *23*, 4466

**DOI:** 10.3390/ijms241512346

**Published:** 2023-08-02

**Authors:** Alecio F. Lombardi, Yajun Ma, Hyungseok Jang, Saeed Jerban, Qingbo Tang, Adam C. Searleman, Robert Scott Meyer, Jiang Du, Eric Y. Chang

**Affiliations:** 1 Research Service, Veterans Affairs San Diego Healthcare System, San Diego, CA 92161, USA; q1tang@health.ucsd.edu (Q.T.); ericchangmd@gmail.com (E.Y.C.); 2 Department of Radiology, University of California San Diego, San Diego, CA 92161, USA; yam013@health.ucsd.edu (Y.M.); h4jang@health.ucsd.edu (H.J.); sjerban@health.ucsd.edu (S.J.); asearleman@health.ucsd.edu (A.C.S.); jiangdu@health.ucsd.edu (J.D.); 3 Orthopaedic Surgery Service, Veterans Affairs San Diego Healthcare System, San Diego, CA 92161, USA; robert.meyer3@va.gov

In the original publication, there was a mistake in Figure 1 as published [1]. The scale of the color bar at the right side of the pH row in both Figure 1A,B should go from 6.5 to 8, instead of 0 to 5. The corrected Figure 1 appears below. The authors state that the scientific conclusions are unaffected. This correction was approved by the Academic Editor. The original publication has also been updated.

## Figures and Tables

**Figure 1:**
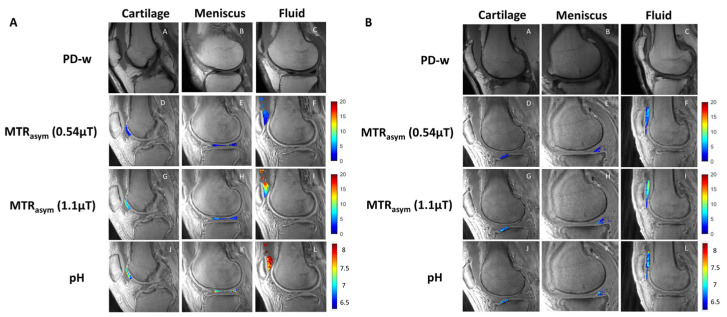
Representative image examples from patients without OA (**A**) and with OA (**B**). Sagittal PD-weighted (first row), low-power acido-CEST UTE (second row), high-power acido-CEST UTE (third row), and pH pixel maps (fourth row) of cartilage, meniscus, and fluid. The pH is directly correlated with the radiofrequency power mismatch (RPM) measurements, as described in Equations (3) and (4). Note the higher pH values (yellow and red colors) in patients without OA compared with patients with OA (blue colors).
